# How Is the Fear of War Impacting Italian Young Adults’ Mental Health? The Mediating Role of Future Anxiety and Intolerance of Uncertainty

**DOI:** 10.3390/ejihpe14040054

**Published:** 2024-03-26

**Authors:** Giorgio Maria Regnoli, Gioia Tiano, Barbara De Rosa

**Affiliations:** Department of Humanities, University of Naples Federico II, Via Porta di Massa 1, 80133 Naples, Italy; giorgiomaria.regnoli@unina.it (G.M.R.); gioiatiano@gmail.com (G.T.)

**Keywords:** fear of war, mental health, future anxiety, intolerance of uncertainty, young adults

## Abstract

The Russian–Ukrainian conflict is affecting mental health even in communities that are not directly involved in the war; added to this is the escalating conflict in the Middle East and its dangerous spread, which brings the war back to the center of the contemporary social and economic horizon. The present study aims to explore the psychological impact of war in a sample of 310 Italian young adults (18–30 years; *M* = 22.0; *SD* = 2.6) while exploring the relationship between Fear of War and psychological distress and evaluating the mediating effects of Future Anxiety and Intolerance of Uncertainty in this relation. Findings highlighted how Fear of War positively and significantly affects Stress, Anxiety, and Depression, and, at the same time, how it fuels both Future Anxiety and Intolerance of Uncertainty. These constructs, in addition to positively affecting the mental health outcomes considered, mediate the relationship between Fear of War and youth psychological distress with a significant indirect effect observed in all three mediation models performed. Finally, significantly higher levels of psychological distress, Fear of War, and Future Anxiety are reported in women than in men. The findings are discussed with reference to the recent literature on the psychological impact of war and on contemporary youth psychological distress, indicating the importance of educational policies and targeted interventions aimed at supporting this target in coping with multiple contemporary collective stressors.

## 1. Introduction

On 24 February 2021, war returned to Europe and added to a global scenario that was already severely compromised by the transversal effects of the COVID-19 pandemic [1], which has particularly affected the most vulnerable evolutionary targets [2,3,4]. After the Second World War, the Russian invasion of Ukraine is among the most tragic events in European history, and it is expected to have serious long-term economic, social, and health consequences [5]. The number of civilian casualties has rapidly exceeded that of the wars in Iraq, Afghanistan, and Vietnam [6], and the Russian bombing has produced a state of emergency over migration due to the displacement of over five million people [7]. The European economy, which has already been tested by the pandemic, has been further affected by the exponential increase in energy prices, raw materials, and the overall cost of living [8,9]. Furthermore, the continuous attacks on the Zaporizhzhya nuclear power plant have brought back fear of an outbreak of a nuclear war, which had subsided with the end of the Cold War, extending the psychological effects of the war well beyond the area limited by the fighting [10]. Adding to this, the conflict between Israel and Hamas reignited on 7 October 2023, and it risks spreading to the entire Middle Eastern area along with its brutal violence, destruction, and global economic consequences, as we are already witnessing in the current crisis in the Red Sea. In short, in the last two years, war has forcefully returned to the center of world attention in an economic, social, health, and psychological scenario already largely compromised by the recent pandemic trauma [11,12]. Against this background, the succession of collective traumatic events is thought to have the potential to generate cumulative effects. In the literature, several effects of war are reported, among which are the alteration of the development of the socioeconomic fabric, the deterioration of community ties, and the fundamental need for security, protection, and belonging [13,14]. In addition, war also brings economic–financial transformations, the impoverishment of populations, and an increase in malnutrition [15], as well as important consequences in terms of mental suffering [13]. Several studies have highlighted that the direct experience of war generates an increase in anxiety, depression, sleep disorders, and post-traumatic, psycho-somatic symptoms [16,17,18,19,20]. Regarding the Russian–Ukrainian war specifically, the negative impact on the mental health of the population and, more particularly, of adolescents and young adults has already been highlighted by several previous studies [21,22,23,24].

The destructiveness of war, however, can overcome geographical borders and upset the stability of entire continents [25] and, in terms of emotional suffering and distress, it can also impact individuals and communities that are not directly involved in it, as highlighted in a cross-cultural study that compared the situation in Ukraine with that in Poland and Taiwan [26]. Moreover, through television news and social media, media bombardment plays an important role [27] given that people are daily subjected to distressing images from multiple war zones. In the Italian context, young people are particularly hyperexposed to this phenomenon, as they are very active on social networks and use them to seek information on this conflict [28]. The literature also reports that daily exposure to images and information that are too distressing can fuel states of uncertainty and fear [27,29], as well as depressive, anxious, and post-traumatic symptoms even in subjects who are not directly involved [18,26,30], as already highlighted with reference to other potentially traumatic collective phenomena, including the recent pandemic [31,32]. Thus, in a historical moment in which youth distress has been reported to be growing for some time [33,34] and has been particularly increased by the pandemic trauma—so much so that, in Italy, there is talk of a youth emergency in terms of mental health [2,3,4,35]—war risks becoming a further potentially traumatic element that adds to specific contemporary sources of unease [36,37].

Fear is a basic emotion aroused by events and situations that are threatening or perceived as such [38]; it is a physiological state that can be activated involuntarily, but it can also be a conscious mental process [39]. It is a crucial emotion for surviving in dangerous situations [40], but, if excessive and associated with an uncontrollable threat, it can fuel anxiety, stress, and avoidant behavior [41,42]. Boehnke and Schwartz [43] explored and introduced the specific construct of Fear of War, investigating it in relation to trait anxiety, personal values, and beliefs, but not in relation to negative emotions or mental health. Other studies have investigated the relationship between Fear of War and the worsening of mental health in terms of anxiety, depression, and psychosomatic symptoms, especially in adolescents and young adults, reporting a particular vulnerability in female individuals [43,44,45,46]. Starting from the study by Lybarger [47], which highlighted the presence of Fear of War even in populations that are geographically distant from armed conflicts, other researchers have investigated the presence of this construct in different parts of the world and its impact on the mental health of individuals and communities [22,48,49,50].

Thus, it is now evident that, like all collective traumatic events, war impacts psychological well-being, fueling worries and anxieties, but also the fear of the unknown and a sense of uncertainty [10,51,52,53]. These complex mental states are associated with the dispositional component of Intolerance of Uncertainty, which is defined as a set of cognitive, emotional, and behavioral responses that the individual implements to cope with ambiguous and uncertain daily situations [54,55]. This construct expresses “the tendency to be bothered or upset by the (as yet) unknown elements of a situation, whether the possible outcome is negative or not” [56] (p. 6). Intolerance of Uncertainty can also be increased by the lack of salient or sufficient information to understand an ambiguous situation [57] and, as an unpleasant experience, it can trigger the dysfunctional search for information or avoidant behavior that aims to reduce the discomfort through an illusory desire for control [56]. This is understood as a disposition capable not only of influencing how individuals interpret present and future events [58] but also of fueling anxiety and fear [59], becoming a nuclear factor in many psychopathological conditions, including obsessive-compulsive, and generalized anxiety disorders, depression, and eating and post-traumatic behavior disorders [60]. Gullo et al. [61] recently investigated this construct in relation to the pandemic trauma, highlighting that Intolerance of Uncertainty partially mediated between fear of contagion and anxiety, depression, and stress, resulting in a vulnerability factor in coping with the pandemic. Other studies also highlighted its role in reducing psychological well-being during pandemics, predicting the fear of COVID-19, and modulating levels of loneliness [62,63].

In Italy, the current proliferation of war outbreaks has spread the fear of a catastrophic global conflict [64], heightening anxieties and worries, particularly in young adults [28,35], and it could also be affecting their representation of the future. Previous research has long reported how the representation of the future in young adults (which plays a crucial role in the construction of one’s life path [65]), has become negative, distressing, and even dystopian [66,67]. Future Anxiety refers to an attitude toward the future in which negative cognitive and emotional processes prevail over positive ones and fear is stronger than hope [68,69]. This construct has been investigated with regard to the recent pandemic, highlighting its impact on mental health [70,71].

However, only a few studies have taken into consideration its relationship with war, and those that did so exclusively referred to geographic contexts that are directly involved in armed conflicts, stressing how Future Anxiety affects various forms of psychological distress influences mental well-being, and triggers the compulsive search for information and news online in young adults [67,72,73].

### Aim and Hypotheses of the Study

Considering the above literature review on the topic and what has emerged in other cultural contexts [22,48,49,50], the present study aims to explore whether and to what extent Fear of War is affecting Italian young adults’ mental health (Stress, Anxiety, and Depression). At the same time, considering the role that Future Anxiety and Intolerance of Uncertainty have played in other traumatic events [70,72], we decided to investigate their impact in mediating the relationship between Fear of War and psychological distress. This research design arises from the ever more topical need to explore the impact of traumatic collective events—in this case, war—on young adults’ mental health to further investigate the psychological indirect effect of war on mental health in Italy on the one hand, and, on the other, to motivate health and education professionals in developing and implementing intervention programs that support the target audience for this study. 

The recent literature on the topic and the constructs presented in this study—as previously reported—have guided the formulation of the following hypotheses: first, we hypothesized that Fear of War correlated with youth psychological distress (H_1_) and that women would report higher levels of Stress, Anxiety, Depression, and Fear of War than men (H_2_); further, we hypothesized that Fear of War would affect levels Stress, Anxiety, and Depression (H_3_) and would play a significant role in positively fueling Future Anxiety (H_4_) and Intolerance of Uncertainty (H_5_); finally, we assumed that Future Anxiety and Intolerance of Uncertainty would mediate the relationship between Fear of War and psychological distress (H_6_).

The latter hypothesis was inspired by studies that investigated Future Anxiety and Intolerance of Uncertainty as risk factors for mental health in populations that were directly exposed to war contexts or the COVID-19 pandemic [59,60,61,62,63,72,73]. However, to our knowledge, no study has currently explored the relationship between these variables in communities that are not directly involved in a war, particularly in the Italian context where the constructs of Future Anxiety and Fear of War have only recently been introduced in the literature [50,68].

**Figure 1:**
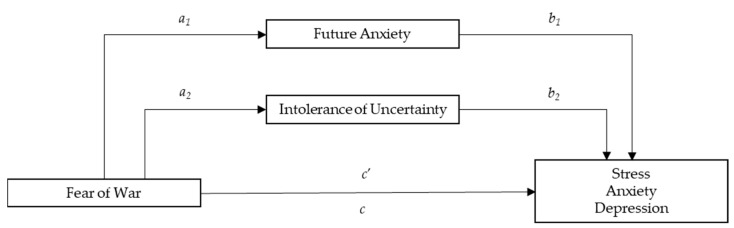
Fear or War: predictor variable (X); Future Anxiety: Mediator 1 (M_1_); Intolerance of Uncertainty: Mediator 2 (M_2_); Stress, Anxiety, and Depression: outcome variables (Y); *c*’ = direct effect of Fear of War on Mental Health (H_3_); *c* = total effect of Fear of War and Mediators on Mental Health. *a*_1_ = effect of Fear of War on Future Anxiety (H_4_); *a*_2_ = effect of Fear of War on Intolerance of Uncertainty (H_5_); *b*_1_ = effect of Future Anxiety on Mental Health; *b_2_* = effect of Intolerance of Uncertainty on Mental Health; *a*_1_*b*_1_ = specific indirect effect of Fear of War on Mental Health through Future Anxiety; *a*_2_*b*_2_ = specific indirect effect of Fear of War on Mental Health through Intolerance of Uncertainty; *a*_1_*b*_1_ + *a*_2_*b*_2_ = Total indirect effect (H_6_).

Hypotheses from 3 to 6 are graphically represented in Figure 1. 

## 2. Materials and Methods

### 2.1. Participants and Procedure

Participants were recruited in Italy between January and May 2023 and all data were collected through self-report questionnaires using Google Forms. Participants were recruited using convenience and snowball sampling methods according to the following criteria: age between 18 and 30 years old, Italian nationality, and residence in Italy. Those who did not fit the criteria and did not give consent were excluded. An initial group of 60 Italian young adults was recruited to reduce the selection bias associated with the sampling methods. The objective of the study and the research protocol were shared within the social spaces of the University of Naples with the group. The group members were then asked to share the questionnaire within their social network. At the same time, the questionnaire was also shared on social media.

The sampling process was preceded by an a priori analysis of the minimum sample size using G*Power. A total of 164 participants were indicated for a medium-size effect (*f*^2^ = 0.15) with 99% power and an alpha of 0.01 (two tails) using linear multiple regression, fixed model, and *R*^2^ increase. We planned to recruit a sample of more than 164 participants to obtain more than sufficient power considering additional mediation effects. All participants included in the study signed a consent form on the first page of the survey that included detailed information about the aim and procedures of the study and the anonymity of the responses.

The sample consisted of 310 Italian young adults, including 158 females (51.0%) and 152 males (48.4%). Participants’ age ranged between 18 and 30 (*M* = 22.0; *SD* = 2.6). Most participants lived in South Italy (87.1%) and overall, 132 (42.6%) declared to live in the city and 178 (54.4%) in the province (rural areas, small villages). Concerning the relationship status, 161 participants (51.9%) were single, 144 (46.5%) were in a non-cohabiting relationship and 5 (1.6%) were in a cohabiting relationship. Regarding the levels of education, 235 participants (75.8%) had completed secondary school, 40 (12.9%) had a bachelor’s degree, 27 (8.7%) had a master’s degree, and only 8 (2.6%) had completed the first level of secondary school. Overall, 198 (63.9%) were students, 57 (18.4%), were working students, 15 (15.5%) were workers, and 7 (2.3%) were unemployed. 

### 2.2. Data Collection Tools

*Personal information.* Participants’ socio-demographic characteristics were assessed using an ad hoc questionnaire describing gender, region of residence, type of residence (town or province), relationship status, level of education, and occupation.

The *Fear of War Scale* (FOWARS) [49,50] is a 12-item self-report instrument with a 5-point Likert scale ranging from 1 (Strongly disagree) to 5 (Strongly agree) and measuring the Fear of War across two subscales: the Physiological Dimension of Fear and the Experiential Dimension on Fear. At the same time, it provides a total score—which was used in this study—where values above 2.5 indicate that the participant is very likely to experience Fear of War [49,50]. In the adaptation and validation study, the scale showed good psychometrics proprieties and high internal consistency [50]. In the current study, a total score for Fear of War was used and its Cronbach’s *α* was 0.89.

The *Intolerance of Uncertainty Scale—Short Form* (IUS-12) [58,74] is a 5-point Likert scale ranging from 1 (Strongly disagree) to 5 (Strongly agree) and composed of a two-factor scale that assesses two different subdimensions of intolerance toward uncertainty, namely, “Prospective Intolerance of Uncertainty” and “Inhibitory Intolerance of Uncertainty” [55]. Furthermore, the IUS-12 also provides a total score ranging from 12 to 60 and higher score corresponding to a higher intolerance of uncertainty [75]: this score was used in this study. The authors of the IUS-12 reported good internal consistency [74]. In the current study, Cronbach’s *α* for the overall scale was 0.88.

The *Dark Future Scale* (DFS) [68], a 5-item self-report instrument with a 7-point Likert-type scale ranging from 0 (Definitely untrue) to 6 (Definitely true), assesses Future Anxiety, a construct that includes cognitive and emotional processes in which fear of the future dominates hope. The total range goes from 0 to 30 and higher scores reflect higher levels of Future Anxiety. The authors of the DFS reported good internal consistency [68]. In the present study, Cronbach’s *α* was 0.88.

The *Depression, Anxiety, and Stress Scale* (DASS-21) [76,77] is a 21-item self-report instrument with a 4-point Likert-type scale ranging from 0 (Did not apply to me at all) to 3 (Applied to me very much, or most of the time), which assessed Depression, Anxiety, and Stress in the last 7 days by using three subscales composed of 7 items for Depression, 7 for Anxiety, and 7 for Stress. With respect to the Stress dimension, scores between 0 and 10 indicate normal levels, scores between 11 and 18 mild levels, scores between 19 and 26 moderate levels, scores between 27 and 34 severe levels, and scores between 35 and 42 extremely severe levels. As far as the Anxiety dimension is concerned, scores between 0 and 6 indicate normal levels, scores between 7 and 9 mild levels, scores between 10 and 14 moderate levels, scores between 15 and 19 severe levels, and scores between 20 and 42 extremely severe levels. Finally, the scores of the Depression dimension between 0 and 9 indicate normal levels, those between 10 and 12 mild levels, those between 13 and 20 moderate levels, those between 21 and 27 severe levels, and those between 28 and 42 indicate extremely severe levels. The authors of the Italian version of DASS-21 reported good psychometric properties and good internal consistency [77]. In the present study, Cronbach’s *α* was 0.89 for Depression, 0.89 for Anxiety, and 0.86 for Stress.

### 2.3. Data Analysis Plan

Descriptive analysis, mean, and standard deviation were conducted for all variables, and reliability analysis was performed using Cronbach’s *a*, which was considered good when both overall and dimensional values were greater than 0.70. Kurtosis and Skewness were also evaluated to verify the normal univariate distribution of psychological variables; values ranging from −1.5 to + 1.5 were indicative of a normal distribution of the variables [78]. To test hypothesis 2 (H_2_) and other potential differences in socio-demographic variables, *t*-test and ANOVA analyses were performed (*p* < 0.05). Cohen’s *d* and eta-square (*η*^2^) were used to measure effect sizes.

Correlations were calculated using Pearson’s coefficient (*r*; between 0.10 and 0.29 = small association; between 0.30 and 0.49 = medium association; and >0.50 = large association; *p* < 0.05) to evaluate how age, gender, and psychological variables were associated with each other and to test hypothesis 1 (H_1_). 

A preliminary check for multicollinearity between the independent variables and mediators was carried out by considering values of tolerance greater than 0.1 and Variance Inflation Factor (VIF) smaller than 5.0 as good. The Durbin–Watson values were also verified and considered great if they were near 2 [79].

Three multiple regression analyses were conducted to verify the significance of the variables and the adequacy of the hypothesized selected mediators. In doing so, standardized beta (*β*), *t*-values, and *R^2^* were used (*p* < 0.05). 

To test Hypotheses 3 (H_3_) to 6 (H_6_), parallel mediation analyses were conducted to explore direct effects and specific indirect effects caused by each mediator. PROCESS macro 4.2 for SPSS was used [80] to test these hypotheses. Model 4 was selected to examine multiple mediators in the parallel mediation models. Three parallel mediation models were carried out for Stress, Anxiety, and Depression as outcome variables (Y). In each mediation model, Fear of War was chosen as the independent variable (X), and Future Anxiety (M_1_), and Intolerance of Uncertainty (M_2_) were selected as mediators. Age and Gender were controlled for as covariates starting from the results of correlations, *t*-tests, ANOVA, and regressions. The statistical significance of the total indirect effect of mediating variables (H_6_) was examined using bootstrapping methods to estimate bias-corrected asymmetric confidence intervals (CIs) with 5000 resamples with replacement (95% CIs not inclusive of zero indicate significant effects). With Model 4, the mediators were analyzed simultaneously while controlling for the effect of one another. At the same time, the indirect effects of single mediators produced by PROCESS were further assessed with the Sobel test technique based on a normality assumption (*z* > 1.96; *p* < 0.05).

Statistical analyses were performed with SPSS 29 and AMOS 29 [81].

## 3. Results

### 3.1. Descriptive Statistics and Group Differences

Response range, Means, Standard Deviations, *t*-tests about gender differences in relation to psychological variables, Cronbach’s *a*, Skewness, and Kurtosis are shown in Table 1. The mean of FOWARS Global was 3.17 (*SD* = 0.87), that of DFS was 19.34 (*SD* = 6.75), and that of IUS-12 Global was 35.23 (*SD* = 9.81). The mean for Prospective Intolerance of Uncertainty was 20.45 (*SD* = 6.06) and 14.78 (*SD* = 4.28) for Inhibitory Intolerance of Uncertainty. Finally, the means for Stress, Anxiety, and Depression were, respectively, 25.59 (*SD* = 10.71), 17.71 (*SD* = 10.97), and 20.80 (*SD* = 10.77). Skewness and Kurtosis values indicate that the psychological variables considered had a normal distribution.

As seen in Table 1, *t*-tests showed significant gender differences. Female participants reported higher levels than male ones for FOWARS (*M_F_* = 3.43 vs. *M_M_* = 2.89; *t*_(306)_ = 5.73; *p* < 0.001; *d* = 0.65), DFS (*M_F_* = 17.46 vs. *M_M_* = 21.09; *t*_(306)_ = 4.88; *p* < 0.001; *d* = 0.56), Stress (*M_F_* = 28.5 vs. *M_M_* = 22.47; *t*_(306)_ = 5.59; *p* < 0.001; *d* = 0.64), Anxiety (*M_F_* = 20.40 vs. *M_M_* = 17.73; *t*_(306)_ = 4.70; *p* < 0.001; *d* = 0.54), and Depression (*M_F_* = 22.50 vs. *M_M_* = 18.91; *t*_(306)_ = 2.97; *p* = 0.00; *d* = 0.34). These results supported H_3_. 

ANOVA and post hoc tests also showed significant differences in relation to occupation. Indeed, students reported higher levels than working students for FOWARS (*M_S_* = 3.29 vs. *M_WS_* = 2.95; *F*_(3, 309)_ = 3.71; *p* < 0.01; *η^2^* = 0.03). No further significant group differences were found considering other socio-demographic variables.

### 3.2. Correlations 

Correlations between the age of participants and the psychological variables are presented in Table 2. The results show how the FOWARS correlated positively and significantly with IUS-12 (*r* = 0.27; *p* < 0.01), DFS (*r* = 0.42; *p* < 0.01), Stress (*r* = 0.39; *p* < 0.01), Anxiety (*r* = 0.46; *p* < 0.01), and Depression (*r* = 0.38; *p* < 0.01), thus strongly confirming Hypothesis 2 (H_2_). Age was significantly and negatively correlated with FOWARS (*r* = −0.14; *p* < 0.05), while significantly and positively correlated with Stress (*r* = 0.11; *p* < 0.05) and Anxiety (*r* = 0.12; *p* < 0.05). Gender was significantly and positively correlated with FOWARS (*r* = 0.32; *p* < 0.01), DFS (*r* = 0.27; *p* < 0.01), Anxiety (*r* = 0.27; *p* < 0.01), and Depression (*r* = 0.18; *p* < 0.05), while it was significantly and negatively correlated with Stress (*r* = −0.30; *p* < 0.01).

### 3.3. Preliminary Assumptions and Regression Analyses

Regarding preliminary assumptions, the tolerance values varied between 0.66 and 0.97, Variance Inflation Factor (VIF) values ranged from 1.03 to 1.51 and the Durbin–Watson Values, evaluated for the three models on mental health outcomes, ranged from 1.91 to 2.10, indicating that multicollinearity and residual problems were not present. 

Three multiple regression models were performed to judge whether to include them in the path model and to verify the significance of the variables. The results—shown in Table 3—indicated that all mediators could be included in parallel mediation models.

### 3.4. Results of Parallel Mediation Models on Stress, Anxiety, and Depression

**Figure 2:**
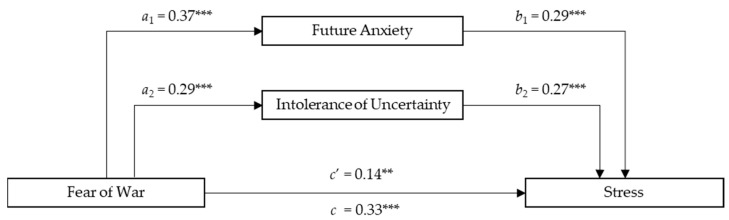
The mediating effects of Future Anxiety and Intolerance of Uncertainty between Fear of War and Stress. Description: *N =* 310, ** *p* < 0.01; *** *p* < 0.001 All present effects are standardized; Control Variables: Age, Gender; *a*_1_ = effect of FOWARS on DFS; *a*_2_ = effect of FOWARS on IUS-12; *b*_1_ = effect of DFS on STRESS; *b*_2_ = effect of IUS-12 on STRESS; *c*’ = direct effect of FOWARS on STRESS; *c* = total effect of FOWARS and Mediators on STRESS.

The total amount of variance accounted for by the overall Stress model was 38% when adjusted for the sample size and the predictor’s number. As presented in Figure 2, the total effect of Fear of War on Stress was significant (*c* = 0.33; *SE* = 0.63; *p* < 0.001), as was the direct effect of Fear of War on Stress (*c’* = 0.14; *SE* = 0.59; *p* < 0.001). 

The direct effect of Fear of War on Future Anxiety was positive and significant (*a*_1_ = 0.37; *SE* = 0.42; *p* < 0.001), as was the direct effect of Fear of War on Intolerance of Uncertainty (*a*_2_ = 0.29; *SE* = 0.66; *p* < 0.001). In addition, participants with higher Future Anxiety (*b*_1_ =  0.29; *SE* = 0.08; *p* < 0.001) and Intolerance of Uncertainty (*b*_2_  =  0.27; *SE* = 0.05; *p* < 0.001) reported higher Stress scores. Findings also showed a significant total indirect effect of Fear of War and Stress via Future Anxiety and Intolerance of Uncertainty (*total indirect effect* = 0.18; *SE* = 0.03; 95% CI [0.12, 0.25]). Considering both confidence intervals and Sobel’s test to assess the significance of a single mediator’s indirect effects, the results showed that the indirect effect of Future Anxiety (*a*_1_*b*_1_ = 0.11; *SE* = 0.02; 95%; CI [0.06, 0.16]; *t_a_*_1_ = 6.69, *t_a_*_2_ = 5.19; *Sobel z* = 4.10; *p* < 0.001) and the indirect effect of Intolerance of Uncertainty (*a*_2_*b*_2_ = 0.08; *SE* = 0.02; 95%; CI [0.04, 0.13]; *t_b_*_1_ = 4.89, *t_b_*_2_ = 5.25; *Sobel z* = 3.57; *p* < 0.001) was significant. Age did not have a significant impact on the total effect model (*β_age_* = −0.5; *p* = 0.34) while Gender was found to be statistically associated with FOWARS in the total effect model (*β_gender_* = 0.19; *p* < 0.001), indicating that females experienced more stress levels in relation to Fear of War compared to males. The findings for the parallel mediation model on Stress with the mediator’s role of Future Anxiety and Intolerance of Uncertainty are presented in Figure 2.

**Figure 3:**
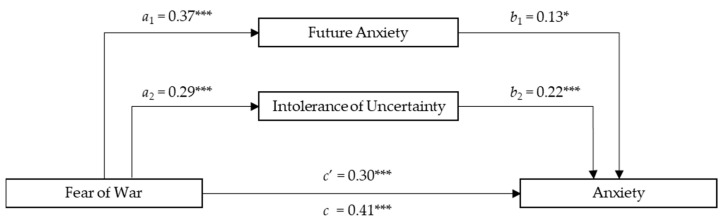
The mediating effects of Future Anxiety and Intolerance of Uncertainty between Fear of War and Anxiety. Description: *N =* 310, * *p* < 0.05; *** *p* < 0.001 All present effects are standardized; Control Variables: Age, Gender; *a*_1_ = effect of FOWARS on DFS; *a*_2_ = effect of FOWARS on IUS-12; *b*_1_ = effect of DFS on ANXIETY; *b*_2_ = effect of IUS-12 on ANXIETY; *c*’ = direct effect of FOWARS on ANXIETY; *c* = total effect of FOWARS and Mediators on ANXIETY.

The total amount of variance accounted for by the overall Anxiety model was 31% when adjusted for the sample size and the predictor’s number. As presented in Figure 3, the total effect of Fear of War on Anxiety was significant (*c* = 0.41; *SE* = 0.67; *p* < 0.001), as was the direct effect of Fear of War on Anxiety (*c*’ = 0.30; *SE* = 0.69; *p* < 0.001) and that of Fear of War on the mediators (see Model 1). In addition, participants with higher Future Anxiety (*b*_1_  =  0.13; *SE* = 0.09; *p* < 0.05) and Intolerance of Uncertainty (*b*_2_  =  0.22; *SE* = 0.06; *p* < 0.001) had higher Anxiety scores. A significant total indirect effect between Fear of War and Anxiety via Future Anxiety and Intolerance of Uncertainty was also found (*total indirect effect* = 0.11; *SE* = 0.03; 95% CI [0.06, 0.17]). Considering Confidence Intervals and the Sobel Test, the findings showed a significant indirect effect of Future Anxiety (*a*_1_*b*_1_ = 0.05; *SE* = 0.02; 95%; CI [0.01, 0.11]; *t_a_*_1_ = 6.69, *t_a_*_2_ = 2.28; *Sobel z* = 2.16; *p* = 0.03) and Intolerance of Uncertainty (*a*_2_*b*_2_ = 0.06; *SE* = 0.02; 95%; CI [0.02, 0.12]; *t_b_*_1_ = 4.89, *t_b_*_2_ = 4.03; *Sobel z* = 3.11; *p* = 0.002). Age did not have a significant impact on the total effect model (*β_age_* = −0.05; *p* = 0.35) while Gender was found to be statistically associated with FOWARS in the total effect model (*β_gender_* = 0.13; *p =* 0.01), indicating that females experienced more Anxiety levels in relation to Fear of War compared to males. The findings for the parallel mediation model on Anxiety with the mediator’s role of Future Anxiety and Intolerance of Uncertainty are presented in Figure 3.

**Figure 4:**
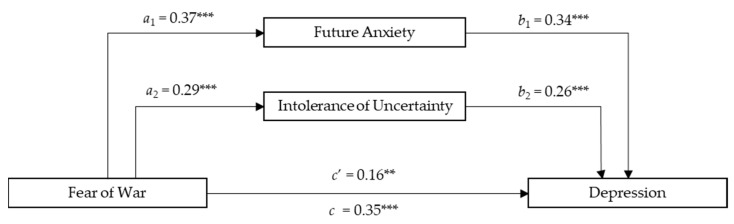
The mediating effects of Future Anxiety and Intolerance of Uncertainty between Fear of War and Depression. Description: *N =* 310, ** *p* < 0.01; *** *p* < 0.001 All present effects are standardized; Control Variables: Age, Gender; *a*_1_ = effect of FOWARS on DFS; *a*_2_ = effect of FOWARS on IUS-12; *b*_1_ = effect of DFS on DEPRESSION; *b*_2_ = effect of IUS-12 on DEPRESSION; *c*’ = direct effect of FOWARS on DEPRESSION; *c* = total effect of FOWARS and Mediators on DEPRESSION.

The total amount of variance accounted for by the overall Depression model was 37% when adjusted for the sample size and the predictor’s number. As presented in Figure 4, the total effect of Fear of War on Depression was significant (*c* = 0.35; *SE* = 0.69; *p* < 0.001), as was the direct effect of Fear of War on Depression (*c’* = 0.16; *SE* = 0.64; *p* < 0.01) and that of Fear of War on the mediators (see Model 1). In addition, participants with higher Future Anxiety (*b*_1_ = 0.34; *SE* = 0.09; *p* < 0.001) and Intolerance of Uncertainty (*b*_2_  =  0.26; *SE* = 0.06, *p* < 0.001) had higher Depression scores. A significant total indirect effect between Fear of War and Depression via Future Anxiety and Intolerance of Uncertainty was also found (*total indirect effect* = 0.20; *SE* = 0.03; 95% CI [0.14, 0.27]). Considering Confidence Intervals and the Sobel Test, findings showed a significant indirect effect of Future Anxiety (*a*_1_*b*_1_ = 0.13; *SE* = 0.02; 95%; CI [0.08, 0.18]; *t_a_*_1_ = 6.69, *t_a_*_2_ = 6.05; *Sobel z* = 4.49; *p* < 0.000) and Intolerance of Uncertainty (*a*_2_*b*_2_ = 0.07; *SE* = 0.02; 95%; CI [0.03, 0.13]; *t_b_*_1_ = 4.89, *t_b_*_2_ = 4.97; *Sobel z* = 3.48; *p* < 0.000). Age and Gender did not have a significant impact on the total effect model (*β*_age_ = −0.5; *p =* 0.37; *β*_gender_ = 0.06; *p* = 0.30). The findings for the parallel mediation model on Depression with the mediator’s role of Future Anxiety and Intolerance of Uncertainty are presented in Figure 4.

In all three parallel mediation models presented, Fear of War showed a significant direct effect on Stress, Anxiety, and Depression. These results strongly confirmed hypothesis H_3_. At the same time, Fear of War also showed a positive and significant effect in increasing levels of Future Anxiety and Intolerance of Uncertainty in the participants. These findings confirmed our initial assumptions H_4_ and H_5_.

In all models, the mediation analysis showed that Future Anxiety and Intolerance of Uncertainty mediated the relationship between Fear of War and mental health in parallel, confirming our hypothesis H_6_. Furthermore, mediation analysis showed that both Future Anxiety and Intolerance of Uncertainty significantly mediated the relationship between Fear of War and mental health even when considered individually in all three models (confidence intervals showed no zeros and Sobel tests were always significant with a *p*-value between <0.05 and <0.001). The significant association of Gender with Fear or War in the total effect of the Stress and Anxiety models reinforced previous findings from the *t*-test analyses and further confirmed hypothesis H_2_.

Full summaries of the models with standardized and unstandardized coefficients are shown in Appendix A (Table A1).

## 4. Discussion

Recent studies have highlighted how the Russian–Ukrainian war is generating repercussions on mental health even in the Italian context, fueling, on the one hand, specific concerns associated with the indirect consequences of the war on the economic level and the cost of living [28,51] and, on the other, the levels of Stress, Anxiety, and Depression in the general population, already affected by the recent COVID-19 pandemic [52]. Nonetheless, the indirect psychological impact of war on mental health remains partly unexplored, particularly in young adults. The present study investigated the relationship between Fear of War and youth psychological distress, as well as the impact of the mediating variables of Future Anxiety and Intolerance of Uncertainty on this relationship.

The overall results concerning the outcome variables highlight high psychological distress in young adults, expressed in general severe levels of Stress, Anxiety, and Depression. These are in line with previous studies on the progressive reduction in youth psychological well-being in Italy [33] and confirm the current emergency in terms of mental health [35] and the psychic fragility of young adults, which had already been detected in several parts of the world in studies on the COVID-19 pandemic [3,4,82,83].

The direct effects of the three mediation models presented showed how Fear of War—whose overall levels are above the average values—can positively and significantly predict Stress, Anxiety, and Depression, highlighting that subjects with greater Fear of War tend to have higher levels of psychological distress. Together with the results of the preliminary correlational analyses, these confirm those already found in our adaptation and validation study of the Fear of War Scale, in which this construct was associated with negative mental health outcomes [50]. Thus, the relationship between Fear of War and psychological distress is in line with studies conducted in the past on the impact of Fear of War on mental health in adolescents and young adults [45,46]. Furthermore, it also aligns with the most recent studies conducted on university students involved in war contexts [22] and, in particular, also with those carried out in places that are not directly involved in an armed conflict [48,49]. Considering the studies that reveal the link between media hyperexposure to distressing information/images and mental suffering [18,31] and, in particular, those specifically referring to images of war [27,29], the relationship between Fear of War and Psychological Distress—revealed in this study—could also be associated to the compulsive search for war-related information in young Italian adults [28], probably sustained by the high levels of uncertainty that the war is fueling. Although media hyperexposure was not investigated in the present study, it is a crucial aspect to be integrated into further research. Exploring the association, reported in the literature, between intolerance of uncertainty and media hyperexposure [54] could shed light on the relationship between fear of war and mental health.

To investigate the meaningful relationship between Fear of War and mental health, two mediating variables were considered, viz., Future Anxiety and Intolerance of Uncertainty. Based on our results, both appear to be able to modulate this relationship partially, significantly, and positively, both in parallel and taken individually. Thus, our results suggest that Future Anxiety and Intolerance of Uncertainty constitute risk factors that fuel the impact of Fear of War on the mental health of young adults.

Given that the relationship between Future Anxiety and Fear of War does not seem to have yet been investigated in contexts that are not directly involved in a war, at present it is impossible to compare these results with those of similar studies. Nevertheless, they are supported by other studies conducted on young adults, which highlight that anxiety about the future and the negative representation of the latter constitute risk factors for general malaise and in particular for Stress, Anxiety, and Depression [84,85]. On the contrary, the literature highlights that the ability to maintain a positive representation of the future in the face of sources of uncertainty and various stressors plays a protective function for the definition of identity, planning, and hope, and for a better general mental well-being [65,86]. As regards studies conducted in war contexts, Future Anxiety appears to play a central role in fueling various forms of mental suffering [73]. Furthermore, the original study by Zaleski et al. [87] points out how higher levels of Future Anxiety can implement pessimistic predictions about solutions to complex and global problems, which, specifically in the Italian context, are increasing along with worries and fears about the future of the world [35]. Finally, with reference to the collective traumatic event of the pandemic, Paredes et al. [88] emphasized the role of Future Anxiety as a vulnerability and risk factor that mediated and strengthened the relationship between the perception of the virus as a threat and its impact on mental well-being. The war and its extension constitute another potentially traumatic event of our times, and its threat exacerbates anxieties, fears, and worries in Italian young adults, which appear to be increased by the risk factor of Future Anxiety [28,35].

From the results of our study, it emerges that the other mediator considered—Intolerance of Uncertainty—also plays a significant role in worsening the psychological distress produced by Fear of War. This result is in line with both the studies that highlight how Intolerance of Uncertainty constitutes a vulnerability factor that fuels stress and anxiety, and with those that report the significant relationship between Intolerance of Uncertainty and fear in threatening situations [62,89]. As regards research on collective traumatic events, viz., the specific focus of our research [4,12,50,90,91,92,93], our results appear in line with the studies that not only pinpoint the significant relationship between Intolerance of Uncertainty, the collective traumatic event of the pandemic and mental health [62,63], but above all with the data reported by Gullo et al. [61], according to which Intolerance of Uncertainty strengthened the positive influence of fear of COVID-19 on mental health. The results emerging from our mediation models and, in particular, the positive relationship between Intolerance of Uncertainty and all the negative mental health outcomes considered corroborates those of several clinical studies in which this construct is a central transdiagnostic factor in many disorders of the internalizing sphere [60] and, in general, those highlighted in the literature on the influence that it exerts on how subjects interpret present and future events [57,58], increasing psychological distress in terms of anxiety and fear [59].

According to the data emerging from our *t*-test analyses, confirmed by the mediation models on Stress and Anxiety, women show greater levels of psychological distress than men, as well as higher levels of Fear of War and Future Anxiety. Concerning mental health outcomes, our results are in line with the literature on gender studies, which highlights the greater tendency toward the internalization of problems in women who, for this reason, are more exposed to psychological distress [94]. Our results also reiterate those that emerged in more recent studies on the greater increase in internalizing disorders in young adult women compared to young adult men [95] and those carried out during the pandemic, in which women were found to be more affected by internalizing symptoms, worries, and future anxiety [3,84]. Regarding Fear of War, our results reiterate those found in our previous study [50] and in other recent contributions [48,49], reporting greater levels of both conventional and nuclear Fear of War in women [43,45]. This information could be read starting from the greater female sensitivity for the care and well-being of others that the destructiveness of war leads to [44], but at the same time, it could be a potential response bias supported by cultural stereotypes that may have made it easier for women to recognize and express their emotions [96]. In any case, our results highlight that the female tendency toward internalization, well known in the literature, can function as a risk factor capable of fueling the impact of Fear of War on mental health. 

### Strengths, Limitations, and Directions for Future Research

The strength of the present study consists in the exploration of the indirect psychological impact of war on Italian young adults, in so far as this area of research has not yet been explored in Italy. This investigation was conducted through the analysis of the relationship between Fear of War, Intolerance of Uncertainty, Future Anxiety, and psychological distress in a context that is not directly exposed to conflict. The adoption of parallel mediation models has highlighted how both Future Anxiety and Intolerance of Uncertainty constitute risk factors for mental suffering fueled by Fear of War. This finding could implement the understanding of contemporary youth distress, complementing and enriching specific support interventions.

Despite this, the present study has its limitations. For instance, the use of convenience sampling implies specific biases such as the volunteer bias, which is related to the special characteristics of individuals who voluntarily participate in a study. Another possible bias in the study is linked to the employment of the mono-method since assessing all variables by using self-report instruments may have caused inflation in the observed associations. In addition, the participants are mostly young adult students, which may have influenced our results as well. Future research should try to incorporate more diverse samples, featuring, for instance, more young adult workers.

Taken together, these limitations do not allow for the generalizability of the results obtained to the entire population of Italian young adults. Thus, future research could increase the representativeness of the sample, involve young adults from more diverse areas of Italy, and consider additional variables. 

## 5. Conclusions

The present study investigated the indirect psychological impact of war on mental health in a sample of Italian young adults considering the direct influence of Fear of War on psychological distress on the one hand and, on the other, the mediating effect that Future Anxiety and Intolerance of Uncertainty play in this relationship. The findings suggest that Fear of War positively predicts Stress, Anxiety, and Depression, and the results are in line with recent studies conducted in contexts that are not directly involved in warfare [48,49], including some Italian ones that exclusively explore the general impact of war without using the specific construct of Fear of War [51,52]. Results of parallel mediation models show that Fear of War is increasing Future Anxiety and Intolerance of Uncertainty levels, significantly affecting psychological distress, and potentiating the impact of Fear of War on Stress, Anxiety, and Depression with a significant indirect effect. 

These findings may innovatively enrich the recent body of literature exploring the direct and indirect psychological impact of war in different contexts. In fact, to our knowledge, no published research has yet investigated the relationship between Fear of War, Future Anxiety, and Intolerance of Uncertainty in communities that are not directly involved in war, especially in the Italian context where the first two constructs were only recently introduced. Future Anxiety and Intolerance of Uncertainty have been investigated in contexts of war and other traumatic events such as pandemics [61,62,70,72], showing that among youths, both are vulnerability and are risk factors that fuel mental suffering, as confirmed in our findings. 

The results of the current study could stimulate psychological research to implement interest in the impact that collective events of traumatic nature can have on mental health, at both the individual and community level. Furthermore, they could enrich the understanding of the underlying causes of youth distress, contributing to the design of interventions aimed at supporting the coping of fears, worries, and anxieties associated with contemporary collective traumatic phenomena such as war. Among them, it seems to us that the construction of an expert-led group workspace in which to communicate worries, thoughts, and fears could stimulate a sharing process supporting the capacity to represent and understand collective traumatic events [14,97]. The discovery of sharing the same worries supports the consolidation of group bonds, which, by opening up the possibility of facing them together, could reduce the sense of helplessness, passivity, and loneliness, as well as the sense of uncertainty aroused by collective events with traumatic potential. Rediscovering oneself as an active individual who is able to manage negative emotions could be a protective factor for the intolerance of uncertainty and future anxiety with their effects on mental health. The group can become a space for transformative work in the direction of a collective elaboration that could support emotional empowerment and more proactive behavior, rekindling the hope and self-efficacy of being able to have an effect on one’s self [98].

## Figures and Tables

**Table 1:** Likert range, Means, Standard Deviations, *t*-tests, Cronbach’s α, Minimum and Maximum, Skewness, and Kurtosis.

		Males (*N* = 152)		Females (*N* = 158)		Gender Differences	Total Sample (*N* = 310)					
Variables	Likert Range	M	SD	M	SD	t (d)	M	SD	Min-Max	*a*	Skew.	Kurt.
FOWARS	1–5	2.89	0.82	3.43	0.83	5.73 *** (0.65)	3.17	0.87	1–5	0.89	0.07	−0.44
DFS	0–6	17.46	7.29	21.09	5.72	4.88 *** (0.56)	19.34	6.75	0–30	0.88	−0.67	0.21
IUS-12	1–5	34.42	9.28	36.08	10.28	1.49 (0.10)	35.23	9.81	12–60	0.88	0.24	−0.42
STRESS	0–3	22.47	9.67	28.56	9.44	5.59 *** (0.64)	25.59	10.00	0–42	0.86	−0.18	−0.67
ANXIETY	0–3	14.73	9.64	20.40	11.39	4.70 *** (0.54)	17.71	10.97	0–42	0.89	0.28	−0.76
DEPRESSION	0–3	18.91	10.88	22.51	10.88	2.97 ** (0.34)	20.80	10.77	0–42	0.89	0.06	−0.84

Notes: FOWARS: Fear of War Scale; DFS: Dark Future Scale; IUS-12: Intolerance of Uncertainty Scale; Stress, Anxiety, and Depression: dimensions of DASS-21; ** *p* < 0.01; *** *p* < 0.001; Min–Max: minimum response range–maximum response range; Skew.: Skewness; Kurt.: Kurtosis.

**Table 2:** Correlations between participants’ age and gender and psychological variables.

	1	2	3	4	5	6	7	8
1. Age	--							
2. Gender	--	--						
3. FOWARS	−0.14 *	0.32 **	--					
4. DFS	−0.09	0.27 **	0.42 **	--				
5. IUS-12	0.03	0.07	0.27 **	0.47 **	--			
6. STRESS	−0.11 *	0.30 **	0.39 **	0.52 **	0.45 **	--		
7. ANXIETY	−0.12 *	0.27 **	0.46 **	0.40 **	0.37 **	0.75 **	--	
8. DEPRESSION	−0.07	0.18 **	0.38 **	0.54 **	0.46 **	0.76 **	0.72 **	--

Notes: * *p* < 0.05; ** *p* < 0.01; FOWARS: Fear of War Scale; DFS: Dark Future Scale; IUS-12: Intolerance of Uncertainty Scale; Stress, Anxiety and Depression: dimensions of DASS-21.

**Table 3:** Multiple Regression Analysis Summary Predicting Stress, Anxiety, and Depression (*N =* 310).

Variables	Stress			Anxiety			Depression		
	*β*	*t*	Sig.	*β*	*t*	Sig.	*β*	*t*	Sig.
Age	−0.10	−2.00	0.05	−0.09	−1.98	0.05	−0.06	−1.23	0.22
Gender	0.15	3.05	0.002	0.11	2.25	0.02	0.01	0.23	0.82
FOWARS	0.14	2.74	0.006	0.30	5.52	<0.001	0.16	3.00	0.00
DFS	0.29	5.19	<0.001	0.13	2.28	0.02	0.34	6.05	<0.001
IUS-12	0.27	5.26	<0.001	0.22	4.03	<0.001	0.26	4.97	<0.001
*Adj. R* ^2^	0.38			0.31			0.37		

Notes: *N* = 310; Method = Enter (Standard Regression); *β =* Standardized coefficients. FOWARS = Fear of War Scale; DFS = Dark Future Scale; IUS-12 = Intolerance of Uncertainty Scale—12; *Adj*. *R*^2^ = Adjusted *R*^2^*.*

## Data Availability

The data that support the findings of this study are available from the corresponding author [B.D.R.], upon reasonable request.

## Appendix A

**Table A1:** Summary of unstandardized and standardized estimates of three parallel mediation models.

	Unstandardized Estimates				Standardized Estimates			
	*Coeff.*	*SE*	*LLCI*	*ULCI*	*Coeff.*	*BootSE*	*LLCI*	*ULCI*
**Mediation Model 1 = Stress**								
Total Effect	3.75	0.63	2.50	4.99	0.33 ***			
Direct Effect	1.63	0.60	0.46	2.81	0.14 **			
- FOWARS → DFS	2.84	0.42	2.00	3.68	0.37 ***			
- FOWARS → IUS	3.23	0.66	1.93	4.53	0.29 ***			
- DFS → STRESS	0.43	0.08	0.26	0.59	0.29 ***			
- IUS → STRESS	0.28	0.05	0.17	0.38	0.27 ***			
Total indirect effect	2.11	0.41	1.34	2.95	0.18	0.03	0.12	0.25^#^
- Through DFS	1.21	0.31	0.66	1.87	0.11 ***	0.02	0.06	0.16^#^
- Through IUS	0.90	0.26	0.41	1.44	0.08 ***	0.02	0.04	0.12^#^
Total Model Summary on Stress: *R*^2^ = 0.38; *F*(_5,304_) = 37.29; *p* < 0.000								
**Mediation Model 2 = Anxiety**								
Total Effect	5.22	0.67	3.89	6.55	0.41 ***			
Direct Effect	3.87	0.69	2.45	5.17	0.30 ***			
- FOWARS → DFS	2.84	0.42	2.00	3.68	0.37 ***			
- FOWARS → IUS	3.23	0.66	1.93	4.53	0.29 ***			
- DFS → ANXIETY	0.22	0.09	0.03	0.40	0.13 *			
- IUS → ANXIETY	0.25	0.06	0.13	0.37	0.22 ***			
Total indirect effect	1.41	0.36	0.75	2.16	0.11	0.03	0.06	0.17 ^#^
- Through DFS	0.61	0.29	0.07	1.21	0.05 *	0.02	0.01	0.11 ^#^
- Through IUS	0.79	0.29	0.31	1.42	0.06 **	0.02	0.02	0.12 ^#^
Total Model Summary on Anxiety: *R*^2^ = 0.31; *F*(_5,304_) = 27.59; *p* < 0.000								
**Mediation Model 3 = Depression**								
Total Effect	4.40	0.69	3.03	5.77	0.35 ***			
Direct Effect	1.94	0.64	0.67	3.22	0.16 **			
- FOWARS → DFS	2.84	0.42	2.00	3.68	0.37 ***			
- FOWARS → IUS	3.23	0.66	1.93	4.53	0.29 ***			
- DFS → DEPRESSION	0.54	0.09	0.36	0.72	0.34 ***			
- IUS → DEPRESSION	0.29	0.06	0.17	0.40	0.26 ***			
Total indirect effect	2.46	0.45	1.62	3.41	0.20	0.03	0.14	0.27 ^#^
- Through DFS	1.53	0.32	0.94	2.21	0.13 ***	0.02	0.08	0.18 ^#^
- Through IUS	0.92	0.29	0.40	1.55	0.07 ***	0.02	0.03	0.13 ^#^
Total Model Summary on Depression: *R*^2^ = 0.37; *F*(_5,304_) = 35.47; *p* < 0.000								

Note: * *p* < 0.05 ** *p* < 0.01; *** *p* < 0.001; In the text, standardized indirect effects are also reported; For indirect effects: significance is given by the absence of zeros in the confidence intervals (indicated with #), and * indicates the significance of Sober Test z.
